# Cognitive Performance Patterns in Healthy Individuals with Substantia Nigra Hyperechogenicity and Early Parkinson’s Disease

**DOI:** 10.3389/fnagi.2016.00271

**Published:** 2016-11-15

**Authors:** Rezzak Yilmaz, Susanne Gräber, Benjamin Roeben, Ulrike Suenkel, Anna-Katharina von Thaler, Sebastian Heinzel, Florian G. Metzger, Gerhard W. Eschweiler, Walter Maetzler, Daniela Berg, Inga Liepelt-Scarfone

**Affiliations:** ^1^Hertie Institute for Clinical Brain Research, Department of Neurodegeneration, University of TuebingenTuebingen, Germany; ^2^German Center for Neurodegenerative DiseasesTuebingen, Germany; ^3^Department of Neurology, Christian-Albrechts-UniversityKiel, Germany; ^4^Department of Psychiatry and Psychotherapy, University of TuebingenTuebingen, Germany; ^5^Geriatric Center at the University Hospital of TuebingenTuebingen, Germany

**Keywords:** substantia nigra hyperechogenicity, Parkinson’s disease, cognition, transcranial sonography, prodromal Parkinson’s disease

## Abstract

**Introduction:** Hyperechogenicity of the substantia nigra (SN+) is a risk marker for Parkinson’s disease (PD) which can be detected before the diagnosis. In healthy individuals, SN+ has been associated with slight deficits in specific cognitive functions, suggesting cognitive impairment as a possible pre-diagnostic marker for PD. However, the pattern of cognitive deficits associated with SN+ has not yet been compared with those present in PD.

**Methods:** Data of 262 healthy individuals with normal echogenicity (SN-) and 48 healthy individuals with SN+ were compared with 82 early stage PD patients using the “Consortium to Establish a Registry for Alzheimer’s disease” test battery. First, the test clusters (factors) were identified using a principal component analysis (PCA). Mean group performance of cognitive tests belonging to distinct factors, according to the PCA, and single subtest performances were compared using analyses of variance. Second, the number of individuals with abnormal cognitive performances (*z*-score < -1.0) were compared between groups.

**Results:** Verbal memory, semantic and executive function, and praxis were identified as components of cognitive performances. The SN+ group performed significantly worse than the SN- group in tests assessing semantic and executive function, with a non-significant decrease in verbal memory. On the subtest level, individuals of the SN+ group scored significantly lower than the SN- group on the Boston Naming Test (BNT; *p* = 0.008). In all subtests, the percentages of PD patients with values below the cut-off for abnormal performance were higher than in the SN- group. Moreover, more individuals from the SN+ group scored below the cut-off in the BNT (SN- = 8.4%, SN+ = 20.8%, *p* = 0.01) and TMT-B (SN- = 6.9%, SN+ = 16.7%, *p* = 0.02), compared to the SN- group.

**Conclusion:** This study confirms poorer performance of healthy individuals with SN+ compared to SN- in specific cognitive domains. However, against the SN- group, the cognitive profile of the SN+ group was not fully consistent with the profile of early PD patients. Our data argues that cognitive impairment associated with SN+ might differ slightly from that seen in early PD. Compensational mechanisms in the early phases of neurodegeneration, and the fact that only a subgroup of SN+ will develop PD, may partly explain these differences.

## Introduction

Idiopathic Parkinson’s disease (PD) is clinically diagnosed according to the presence of the cardinal motor symptoms. However, in accordance with the pathological degenerative process that starts many years before clinical diagnosis can be made, several non-motor and unspecific early motor symptoms may be observed. This very early neurodegenerative phase is termed prodromal, or pre-diagnostic, PD (Berg et al., 2015). Well known prodromal non-motor symptoms are hyposmia, REM sleep behaviour disorder (RBD), depression and autonomic dysfunction (Berg et al., 2015), but the role of slight impairment in cognition in the prodromal phase is not yet entirely clear. In the clinical course of PD, cognitive deficits have been shown not only to be a marker of late stages, but may also occur in earlier phases of the disease (Foltynie et al., 2004; Muslimovic et al., 2005; Broeders et al., 2013a). Interestingly, first studies on this matter indicate that cognitive dysfuction may even occur in the prodromal stage (Postuma et al., 2012; Pont-Sunyer et al., 2015; Schrag et al., 2015; Chahine et al., 2016).

Increased echogenicity of the substantia nigra (SN+), visualized by transcranial sonography (TCS) has been demonstrated to increase the risk for future PD diagnosis (Berg et al., 2013a). This ultrasound marker can be identified by experienced sonographers in individuals with a sufficient temporal bone window. At the group level, SN+ in still healthy individuals has previously been shown to be associated with slight impairments in cognition (Liepelt et al., 2008; Hoeppner et al., 2009). As SN+ increases the risk for PD (more than 20-fold in individuals older than 50 years of age, when followed for 5 years) (Berg et al., 2013a), it seems plausible that cognitive deficits in individuals with SN+ may present a specific pattern typical for premotor PD. Moreover, a continuum in the type and degree of cognitive decline in the different stages of PD (prodromal – early – mid – late) seems likely. But, this assumption has never been tested. We therefore decided to take the first part of these stages and compared the cognitive performance between groups of low (SN-) and high (SN+) echogenicity (with the latter ones supposedly at higher risk for PD) and a group of early PD patients, in order to identify a specific pattern of cognitive deficits in elderly with increased risk of future PD.

## Materials and Methods

The study was approved by the ethical committee of the Medical Faculty of the University of Tübingen, Germany. All procedures were in accordance with the Declaration of Helsinki, and informed consent was obtained from all participants.

### Participants

Data of 427 healthy elderly individuals from the “Prospective evaluation of Risk factors for Idiopathic Parkinson’s Syndrome” (PRIPS) study (Berg et al., 2013b), who were later included and followed in the “Tübinger Evaluation of Risk Factors for Early Detection of Neurodegeneration” (TREND) study^1^, were analyzed. This cohort was chosen, as these individuals were recruited without screening for any prodromal markers, so as to avoid the overrepresentation of prodromal PD (individuals primarily from large companies who were older than 50 years of age were asked to participate) (Berg et al., 2013b).

**FIGURE 1:**
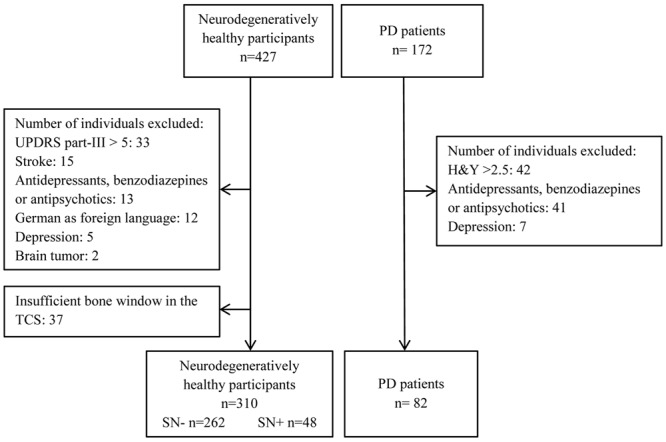
**Flowchart of the study population showing the exclusion criteria**.

Through the outpatient clinic of the Department of Neurology, University of Tübingen, 172 elderly (older than 50) patients with PD were recruited. Patients with PD were diagnosed according to the United Kingdom Brain Bank Criteria. All patients were receiving optimal medication for their symptoms. Only patients with a Hoehn and Yahr Score of ≤2.5 were included into the present analysis, in order to characterize the group of PD as early stage. Other exclusion criteria for all participants were as follows: Moderate or severe depression defined by a score of or above 18 in the “Beck Depression Inventory,” manifest dementia defined by a score in Mini-Mental Status Examination (MMSE) ≤24, mother tongue other than German, history of stroke, brain tumor or medication that could interfere with test performance. For healthy individuals a score of >5 in the motor part of the Unified Parkinson’s Disease Rating Scale (UPDRS) was interpreted as indicative of parkinsonism. Those persons were also excluded from data analysis (see **Figure 1**).

### Transcranial Sonography

The status (SN-, SN+) of the healthy participants was defined by measuring the area of increased echogenicity at the anatomical site of the substantia nigra in the hypoechogenic brainstem using TCS. A hyperechogenic area larger than 0.22 cm^2^ on either side, which corresponded to the 90th percentile of the TREND cohort, was considered as SN+. For details of the examination of substantia nigra using TCS and specific cut-off values, see (Berg et al., 2006; Walter and Skoloudik, 2014).

### Cognitive Assessment and Scores

The following subtests of the German version of the “Consortium to Establish a Registry for Alzheimer’s disease” (CERAD) plus battery were administered (Morris et al., 1988): Verbal Fluency (semantic fluency), Verbal Memory Test (word list learning, delayed recall, and recognition), modified Boston Naming Test (BNT), Visuo-constructional Praxis (Total figure drawing and delayed figure recall), and the Trail Making Test (TMT) parts A and B.

Raw scores of the tests were converted to age, sex, and education-corrected *z*-scores, as provided in the CERAD manual (Aebi, 2002). For the comparison of abnormal cognitive performances (explained below), a *z*-score below -1.0, representing a score lower than one standard deviation (SD) from the mean performance of the normal population, was defined as low performance in each subtest. In general, the cut-off for the definition of low performance can be defined by considering the nature of the analyzed cohort or desired sensitivity/specificity. For instance, mild cognitive impairment (MCI) in PD has been defined as a domain score lower than *z* = -1.5 (Aarsland et al., 2010). In this study, we chose a more liberal cut-off value to identify slight deficits in cognitive performance that could be expected in healthy individuals who do not yet have MCI, but who may be in the early stages of an ongoing neurodegenerative process (Lezak, 2012).

### Statistical Analyses

Analyses of cognitive performances were performed in an explorative manner for the purpose of identifying a common pattern between the risk and PD groups. Statistical analyses of neuropsychological data were performed in two approaches. First, performance of the CERAD subtests was compared between groups; second, comparisons of the percentage of individuals who scored below the defined cut-off (*z* < -1.0) were conducted. Group differences in demographical variables (sex, age, and education) were analyzed using a χ^2^ or a one-way analysis of variance (ANOVA). As part of the first approach, a principal component analysis (PCA, oblique rotation) was performed to define the clusters (factors) of the CERAD subtests, interpreted as cognitive domains. After defining the factors according to the PCA, mean *z*-values of those CERAD subtests with the highest loading on one factor were calculated and compared between study groups by using an ANOVA. Between-group performances in each single CERAD subtest (dependent variables) were also analyzed by an ANOVA with group membership (SN-, SN+, and PD) as the independent factor and, when significant, were followed by subsequent *post hoc* tests of Bonferroni or Games-Howell as appropriate. Correlations between the cognitive tests and the total (right + left) area echogenicity in non-PD groups were also performed using the Pearson’s correlation. In the second approach, χ^2^ tests were performed within groups after re-coding the CERAD test results (continuous variable) into dichotomous categorical variables as higher or lower than the cut-off score (*z* = -1.0, abnormal performance). SPSS Statistics 22.0.0 (SPSS Ltd., Chicago, IL, USA) was used for statistical analyses.

## Results

### Characteristics of Study Groups

An overview of participants excluded from data analysis is given in **Figure 1**. In total, data of 392 participants was analyzed (see **Table 1** for details of demographics). Of those, individuals with SN+ (*n* = 48) and PD (*n* = 82) were significantly older than persons with SN- (*n* = 262, *p* < 0.001). Members of both SN groups had a higher level of education than PD patients (*p* = 0.002). Percentage of males did not differ statistically between study groups.

**Table 1:** Demographical characteristics of the groups.

	SN-	SN+	PD	*P*-value
*N*	262	48	82	
Age (mean ± SD)	63.6 (5.5)	66.3 (5.9)	67.0 (6.5)	<0.001^a∗^
Male, *n* (%)	171 (65)	37 (77)	54 (66)	0.27^+^
Education, years	14.1	14.0	12.8	0.002^a∗^

*N, number; SD, standard deviation; SN, substantia nigra. ^∗^*P*-value significant. ^a^One-way ANOVA. ^+^χ^2^ test.*

### Principal Component Analysis

The PCA was conducted on nine neuropsychological tests (see **Table 2**) with oblique (related) rotation (oblimin). The Kaiser–Meyer–Olkin (KMO) measure verified the sampling adequacy for the analysis (KMO = 0.71). Bartlett’s test of sphericity (χ^2^(36) = 797.69, *p* < 0.001) indicated that correlations between items were sufficiently large for PCA. Three components had eigenvalues over Kaiser’s criterion of 1 and, in combination, explained 60.5% of the variance. **Table 2** shows the factor loadings after rotation. According to the items that gather on the same factors, factor 1 was assigned as verbal memory (31.6% of variance explained), factor 2 as semantic and executive function (15.6% of variance explained) and factor 3 as praxis (13.3% of variance explained).

**Table 2:** Loadings of the principal component analysis showing a three-factor model for cognitive domains.

Neuropsychological test	Component 1 verbal memory	Component 2 semantic and executive function	Component 3 praxis
Boston naming test		0.55	
Semantic fluency		0.53	
Word list learning	0.82		
Word list delayed recall	0.87		
Word list recognition	0.72		
Figure drawing			0.86
Delayed figure recall			0.85
Trail making test-A		0.81	
Trail making test-B		0.80	
Explained variance	31.6%	15.6%	13.3%

### Domain and Subtest Performances of SN-, SN+, and PD Groups

First, the mean *z*-scores calculated for each factor were compared between groups (**Table 3**). PD patients had significantly lower mean *z*-scores in verbal memory (factor 1) and praxis tests (factor 3) than SN-, but not in tests assessing semantic and executive function (factor 2). In contrast, individuals with SN+ showed significantly lower mean *z*-scores in tests associated with semantic and executive function (factor 2) than participants with SN- [Welch *F*(2,107) = 4.8, *p* = 0.01, Games-Howell *p* = 0.02], but not in the mean *z*-scores of tests related to verbal memory (factor 1) and praxis (factor 3). Interestingly, mean factor *z*-scores between patients with PD and individuals with SN+ only differed significantly in praxis function (factor 3).

**Table 3:** Comparison the groups in CERAD subtest and factor levels.

Neuropsychological test	SN- (mean ± SD)	SN+ (mean ± SD)	PD (mean ± SD)	*P*-value PD vs SN-	*P*-value PD vs SN+	*P*-value SN+ vs SN-
Factor 1 (verbal memory)^g^	-0.04 (0.7)	-0.15 (0.7)	-0.33 (0.9)	0.02	ns	ns
Word list learning^b^	-0.3 (0.9)	-0.3 (1.0)	-0.49 (1.1)	ns	ns	ns
Word list delayed recall^b^	-0.03 (0.9)	-0.27 (0.8)	-0.29 (1.1)	0.02	ns	ns
Word list recognition^g^	0.22 (0.8)	0.12 (0.9)	-0.22 (1.1)	0.004	ns	ns
Factor 2 (semantic and executive function)^g^	0.29 (0.7)	0.03 (0.6)	0.10 (0.9)	ns	ns	0.02
Boston naming test^g^	0.34 (0.8)	-0.08 (0.9)	-0.05 (1.1)	0.008	ns	0.008
Semantic fluency^b^	0.06 (1.1)	-0.15 (0.8)	-0.36 (1.1)	0.005	ns	ns
Trail making test-A^g^	0.31 (1.1)	0.18 (1.0)	0.07 (1.4)	ns	ns	ns
Trail making test-B^g^	0.46 (1.0)	0.18 (0.9)	0.46 (1.5)	ns	ns	ns
Factor 3 (praxis)^g^	0.28 (0.9)	0.40 (0.9)	-0.30 (1)	<0.001	<0.001	ns
Figure drawing^g^	0.25 (0.9)	0.33 (0.8)	-0.13 (1.1)	0.02	0.02	ns
Delayed figure recall^g^	0.31 (1.1)	0.46 (1.1)	-0.47 (1.4)	<0.001	<0.001	ns

*ns, non-significant; b, *Post hoc* Bonferroni; g, *Post hoc* Games-Howell.*

Comparisons between PD and SN- groups on the subtest level yielded significant results in all but word list learning and TMT tests. The SN+ group performed significantly worse than the SN- group only in the BNT [Welch *F*(2,100) = 8.4, *p* < 0.001, Games-Howell *p* = 0.008]. Contrary to the SN- group, members of the SN+ group performed similar to the PD group on the word list delayed recall test (SN+ mean = -0.27, PD mean = -0.29), although this was not significantly different from the performance of the SN- group. Additionally, no significant correlation was detected between tests results and the total area of echogenicity (Supplementary Table).

### Frequency of Individuals with Values below Cut-Off

**FIGURE 2:**
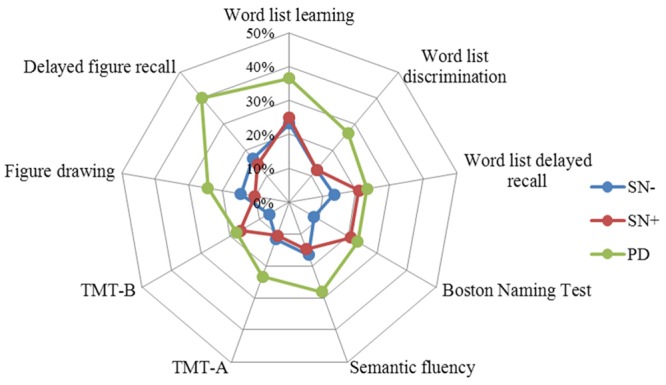
**Percentages of individuals per group with a *z*-score < -1**. Note the increase in the percentage of word list delayed recall test in the SN+ group, together with the Boston Naming Test and TMT-B. TMT, Trail Making Test.

Chi-square tests showed that the percentages of PD patients with a performance below cut-off were significantly higher compared to individuals with SN- in all tests (**Table 4**). In contrast, PD and SN+ deviated significantly only in the delayed figure recall test (*p* = 0.002). The SN+ group had a higher frequency of individuals with abnormal performance in the BNT (SN- = 8.4%, SN+ = 20.8%, *p* = 0.01), and TMT-B (SN- = 6.9%, SN+ = 16.7%, *p* = 0.02) compared to the SN- group. Similar to the mean value comparison, the percentage of individuals who scored below the cut-off in the word list delayed recall test in the SN+ group (20.8%) was close to the PD group (23.2%), although it lacked significance compared to the SN- group (13.4%; *p* = 0.18) (**Figure 2**).

**Table 4:** Percentages of individuals per group with a *z*-score < -1 in the subtests of the CERAD battery.

Groups	SN- (%)	SN+ (%)	PD (%)	*P*-value PD vs SN-	*P*-value PD vs SN+	*P*-value SN+ vs SN-
**Factor 1 (verbal memory)**						
Word list learning	23.3	25.0	36.6	0.017	ns	ns
Word list delayed recall	13.4	20.8	23.2	0.033	ns	ns
Word list recognition	12.6	12.5	26.8	0.002	ns	ns
**Factor 2 (semantic and executive function)**						
Boston naming test	8.4	20.8	23.2	<0.001	ns	0.01
Semantic fluency	16.4	14.6	28.0	0.02	ns	ns
Trail making test-A	11.5	10.4	23.2	0.008	ns	ns
Trail making test-B	6.9	16.7	17.9	0.003	ns	0.02
**Factor 3 (praxis)**						
Figure drawing	14.5	10.4	24.4	0.037	ns	ns
Delayed figure recall	16.8	14.6	40.2	<0.001	0.002	ns

*ns, non-significant.*

## Discussion

In this cross-sectional study, the cognitive performance of healthy individuals with SN+, a risk factor for the later development of PD, and of those without this risk factor (SN-) were compared to patients with early PD using two different statistical approaches. Our results indicate that the percentage of individuals with abnormal test performance (<1 SD below norm mean) was higher in patients with PD compared to the SN- group in all tests. Furthermore, considering that SN+ is associated with the prodromal neurodegenerative process leading to PD, our data support previous findings indicating that cognitive deficits are observed even in the early stages of PD. Performance of individuals with SN+ differed from that of SN-, especially in tests assessing confrontation naming and executive function and – although not significant – in the verbal memory domain. Moreover, the SN+ and PD groups did not differ statistically in memory and language function, and scored similarly in some tests, with the exception of the visual construction domain.

The three clusters of neuropsychological subtest performances that were revealed by the PCA analysis are in accordance with the previously known cognitive domains. Comparing the mean *z*-scores of these clusters, the SN+ group showed intermediate performance, i.e., between SN- and PD groups, in the verbal memory domain (**Table 3**). Factor 2 (semantic and executive function) score was significantly lower in the SN+ compared to the SN- group. Likewise, mean performance of the SN+ group in the BNT also was significantly worse than that of the SN- group. Given that the SN+ group is comprised of individuals some of which may develop PD in the future, slight impairments may have already affected their executive functions and confrontation naming. Previous studies have shown that performance in these domains can be impaired in early PD patients (Poletti et al., 2012; Broeders et al., 2013b; Hanna-Pladdy et al., 2013). Interestingly, although not significant, mean Factor 2, BNT and TMT-B scores were slightly lower in the SN+ group than in the PD group. This suggests that on a group level, individuals with SN+ are slightly more impaired than early PD patients in confrontation naming and executive function. This inconsistency could be explained either by additional factors, such as possible compensation due to the dopaminergic therapy in some patients in the PD group (Kehagia et al., 2010), or by the possibility that the SN+ group is not an ideal representation of premotor PD in all cognitive domains. However, when comparing only the abnormal scores, the PD group had the highest prevalence in all tests (**Table 4**).

Studies on cognitive performance in individuals with SN+ are rare, and, due to the fact that only a minority of the persons involved will develop PD, not as consistent as those in the early PD group. Along with previous studies reporting executive dysfunction in the SN+ group (Liepelt et al., 2008; Hoeppner et al., 2009), our group has in a large cohort recently shown that SN+ can be associated with a slight decline in the word list delayed recall test (Yilmaz et al., 2016) which is in accordance with other reports of memory impairment in early PD (Aarsland et al., 2009; Broeders et al., 2013b; Schrag et al., 2015). With a smaller sample size, the present study did not replicate this finding, but still suggests the tendency of a decreasing performance in the SN+ group, toward the level of PD group performance, both in mean score and low performance comparisons. The lack of a significant correlation between cognitive scores and the echogenic area was not surprising, since the echogenicity of substantia nigra do not to change during the course of the disease (Berg et al., 2005) and only constitutes a risk marker rather than reflecting the disease severity.

Previous studies on cognition in PD risk-groups report decline in several areas. One study found deficits in the MMSE, TMT-A, and constructional praxis in patients with REM sleep behavioral disorder (Youn et al., 2015), whereas another reported impairment in TMT-B in incidental Lewy body disease (Adler et al., 2010). Visual short-term memory task and executive function deficits were also found in healthy individuals with GBA and LRRK mutations (Thaler et al., 2012; Zokaei et al., 2014). Executive dysfunctions have also been shown to be present in the first-degree relatives of PD patients (Dujardin et al., 1999). One recent study reported executive dysfunction in healthy adults with hyposmia and reduced dopamine transporter binding (Chahine et al., 2016). These findings represent a growing body of evidence that suggest a problem in executive functions in PD risk-groups, occuring before the clinical diagnosis of PD. A slight deficit in executive functions was also found to be predictive of PD in a large prospective population-based study (Ross et al., 2012). Some of these studies compared the risk group with healthy controls but did not include PD patients in the analyses. In our study, however, we included a PD group in order to validate the finding in the risk group, and to compare the pattern and degree of cognitive impairment.

The evaluation of cognitive functions in the PD risk-group may be useful (Adler, 2011), but there are several factors that complicate the search for the cognitive profile of pre-diagnostic PD. First of all, PD itself has a heterogeneous cognitive profile (Svenningsson et al., 2012). Secondly, the rate of progression of the premotor markers in the pre-diagnostic phase is unknown (Postuma et al., 2012). Additionally, during the long course between the beginning of neurodegeneration and clinical PD diagnosis, (motor or non-motor) symptoms of PD may occur at different temporal stages (Schrag et al., 2015). Therefore, detecting or following cognitive decline in PD risk-groups is not an easy task. Furthermore, all currently known risk or prodromal factors, with the exception of RBD, are frequently observed in healthy elderly who will never develop PD. For instance, although it is a relatively strong risk factor, the prevalence of SN+ is nearly ten times higher than the prevalence of PD, meaning, that most of the individuals with SN+ will not be diagnosed with PD in their lifetime (Walter, 2011). Therefore, the search for cognitive patterns in the SN+ group, taken as the sole risk factor, may not be adequate for addressing pre-diagnostic cognitive impairment in PD, but may be part of a spectrum of risk factors.

Some limitations of our study need to be mentioned. First, no correction for multiple testing was applied. Second, the PD group did not have a TCS examination and therefore the data regarding the echogenic area in the PD group could not be added to the correlation analysis. Moreover, knowing that the prevalence of the SN+ in PD patients is around 90%, approximately eight patients in PD group could be assumed as having a normal echogenicity, which could additionally explain the lack of perfect overlap between the SN+ and PD groups. Third, the relatively small sample size of the SN+ group (*n* = 48) may have suppressed differences especially compared to the SN- group, which could have been detected at the group level. In summary, this study revealed that although a cognitive difference between the SN groups as well as a stepwise impairment toward the cognitive profile of early PD can be observed in the risk group (SN+) in some subtests, a perfect continuum in the cognitive deficit pattern from healthy via PD risk to early PD could not be demonstrated. In the future, more strictly defined and longitudinally followed risk/prodromal groups stratified by several markers are needed in order to differentiate the cognitive profile of individuals with SN+ that will develop PD, from those of the SN- and non-PD SN+ group. This will help to establish a likelihood ratio of prodromal PD in individuals with specific cognitive profiles. The presence of several other risk and prodromal markers may help to calculate the risk for prodromal PD according to the new research criteria for prodromal PD (Berg et al., 2015). Therefore, the progression rate of the cognitive impairment prior to the diagnosis of PD could then be investigated.

## Author Contributions

RY, IL-S, FM, GE, WM, and DB designed the study, SG, BR, US, A-KvT, and FM acquired and organized the data. RY, SH, and IL-S designed and performed the statistical analysis reviewed by all authors. All authors contributed to the writing and the critique of the manuscript.

## Conflict of Interest Statement

The authors declare that the research was conducted in the absence of any commercial or financial relationships that could be construed as a potential conflict of interest.

## Footnotes

1 http://www.trend-studie.de
