# Supplementary material for: Cognitive Performance Patterns in Healthy Individuals with Substantia Nigra Hyperechogenicity and Early Parkinson’s Disease
Source: Front Aging Neurosci. 2016 Nov 15;8:271. doi: 10.3389/fnagi.2016.00271 (PMC5108760; doi:10.3389/fnagi.2016.00271)
Supplement: Supplementary file 1 [file Table_1.docx]

Supplementary Table: Correlations between the total area of echogenicity and neuropsychological tests.

| Neuropsychological test | Correlation coefficient (r) | P value |
| --- | --- | --- |
| Boston Naming Test | -.07 | 0.26 |
| Semantic fluency | -.06 | 0.33 |
| Word list learning | -.02 | 0.79 |
| Word list delayed recall | -.11 | 0.05 |
| Word list recognition | .08 | 0.18 |
| Figure drawing | .02 | 0.71 |
| Delayed figure recall | .10 | 0.07 |
| Trail Making Test-A | .03 | 0.65 |
| Trail Making Test-B | .03 | 0.57 |

r, Pearson’s correlation coefficient.
